# State-transfer modeling collective behavior of multi-ball Bernoulli system based on local interaction forces

**DOI:** 10.3389/frobt.2022.980586

**Published:** 2022-11-10

**Authors:** Fan Ye, Arsen Abdulali, Fumiya Iida

**Affiliations:** Bio-Inspired Robotics Lab, Department of Engineering, University of Cambridge, Cambridge, United Kingdom

**Keywords:** state-transfer, collective behavior, multi-agent system, Bernoulli-ball system, game theory, swarm robotics

## Abstract

Collective behavior observed in nature has been actively employed in swarm robotics. In order to better respond to external cues, the agents in such systems organize themselves in an ordered structure based on simple local rules. The central assumption, in swarm robotics, is that all agents in the system collaborate to fulfill a common goal. In nature, however, many multi-agent systems exhibit a more complex collective behavior involving a certain level of competition. One representative example of complex collective behavior is a multi-ball Bernoulli-ball system. In this paper, by extracting local force among the Bernoulli balls, we approximated the state-transfer model mapping interaction forces to observed behaviors. The results show that the collective Bernoulli-ball system spent 41% of its time on competitive behaviors, in which up to 84% of the interaction state is unorganized. The rest 59% of the time is spent on collaborative behavior. We believe that the novel proposed model opens new avenues in swarm robotics research.

## 1 Introduction

In the animal kingdom, collective behaviors arise from the local exchange of information between individuals (Couzin, 2009; Ramdya et al., 2015). This circumvents the cognitive limitation of individuals and helps a group of animals like a flock of birds, a school of fish, and a society of ants to respond to environmental cues (Schaerf et al., 2017), and align in an ordered and complex structure (Hooker, 2011). This ability to form a complex shape through a local information exchange is considered to be a primary source of inspiration in robotics self-organization research (Willshaw, 2006; O’Sullivan, 2009; Camazine et al., 2020).

With the growing demand for robot functionalities, scientists strive to develop a large group of robots that cooperate to accomplish complicated tasks (Dorigo et al., 2021). Inspired by self-organization in nature, swarm robots are designed to exhibit robust, scalable, and flexible behaviors through local interactions (Brambilla et al., 2013). Scientists first try to simulate the local information exchange of a flock of birds (Reynolds, 1987; Vicsek et al., 1995), and mathematically proved the emergence of the self-organization process in this system (Jadbabaie et al., 2003). Then, a variety of swarm robots are designed to imitate social animals, e.g., collective bristle robots (Giomi et al., 2013), ant-like cooperating robots (Krieger et al., 2000), particle swarm robots (Li et al., 2019), and collective drones (Vasarhelyi et al., 2018). Some of them interact through physical contact, and others have additional information exchange with a decision-making algorithm to enable them to do locomotion and detection (Olfati-Saber, 2006; Ebert et al., 2020). Without a central leader to send commands to individuals, the interaction rules govern the swarm robots and limit their functionalities.

**FIGURE 1:**
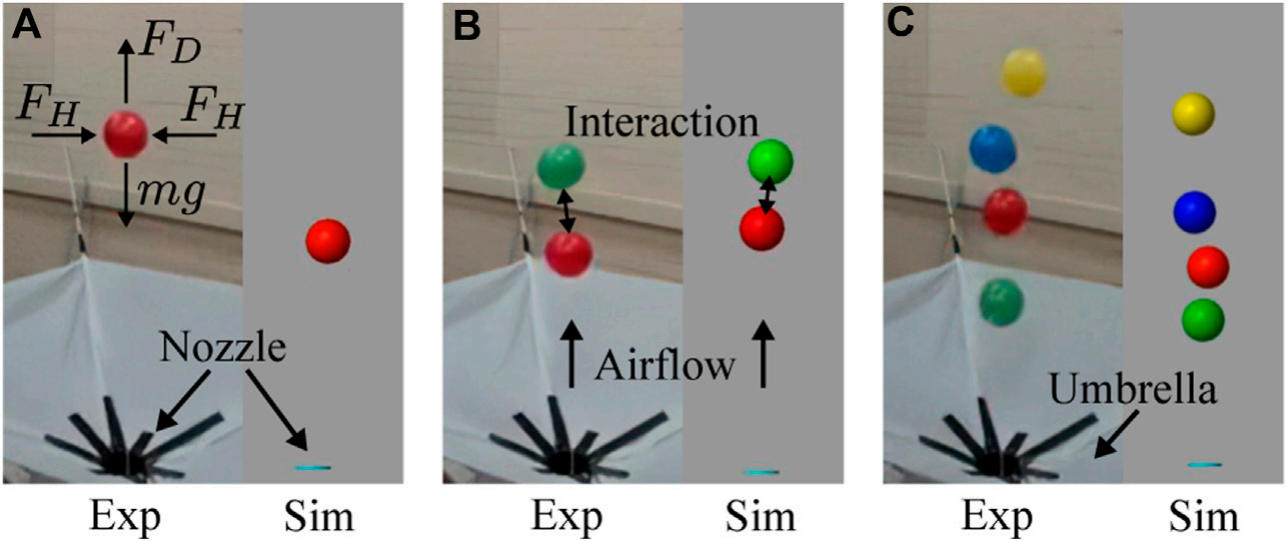
The collective Bernoulli-ball system with 1 **(A)**, 2 **(B)**, and 4 **(C)** balls in experiments and simulation. The vertical airflow comes out of the nozzle to provide drag forces (*F*_*D*_) and horizontal forces (*F*_*H*_) to the balls. Interactions emerge between multiple balls. The umbrella is used to collect falling balls and put them back into the airflow.

To enhance their functionalities, a more intelligent interaction rule should be applied to swarm robots. Usually, scientists in swarm robotics focus on the development of the interaction rules between robots. For example, ant-like swarm robots use radio communication to cooperatively search for objects and for navigation (Hoff et al., 2010). Bee-like swarm robots use a broadcast architecture to form the robot group and produce a specified distribution of pollination (Berman et al., 2011). Fish-like swarm robots communicate through lights to exhibit collective behaviors like synchrony, dispersion, dynamic circle formation, and search-capture (Berlinger et al., 2021). Morphologically communicated swarm robots deform their shape through physical interaction. These researches take the interactions between individuals as a method of communication (Rubenstein et al., 2014; Sharma et al., 2020). However, in nature, especially for birds (Lissaman and Shollenberger, 1970) and fish (Liao et al., 2003), the interactions in the swarm include information exchange and dynamic forces that grant them high coordination complexity. These methods, however, require an intelligent controller to help individual agents make simple decisions. To eliminate the uncertainty of the level of contribution of individuals’ intelligence, we propose to use a Bernoulli-ball system where interactions are governed solely by physical rules. Furthermore, we are interested in the emergence of collaboration and competition through the analysis of dynamic forces. As shown in Figure 1, in this system, a nozzle at the bottom emits vertical airflow, which allows the balls to float in the air (Nudehi et al., 2018; Howison et al., 2020a). When only one ball is put into the system, it gets vertically balanced by compensating the gravity force with the drag of the airflow (Taneda, 1956; Flemmer and Banks, 1986) and horizontally balanced by side forces pointing to the horizontal center governed by Bernoulli’s principle (Massey and Ward-Smith, 2018). When there are multiple balls in the same air stream, the interaction emerges among balloons by influencing the airflow surrounding them (Magarvey and Bishop, 1961; Lee, 1979; Ormières and Provansal, 1999; Tyagi et al., 2006). This interaction leads to competition, where one balloon pushes away the other one from the air stream, and collaboration, when one balloon influences the flow preventing the other one from the fall. Even though it is difficult to observe interaction forces in a physical Bernoulli ball system, they can be approximated in simulation as in our previous work (Ye et al., 2022). However, based on force observations, it is still not trivial to find the relation between force patterns and consequent collective behavior.

**FIGURE 2:**
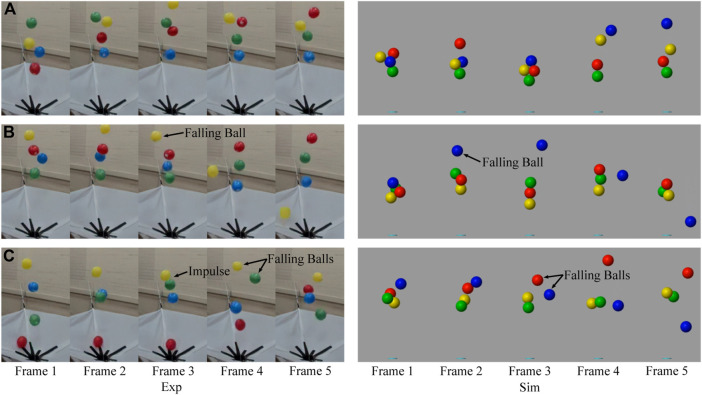
Frame arrays of several typical collective behaviors showing collaboration and competition in both experiment and simulation, like win-win situation **(A)**, kick-out effect **(B)**, and lose-lose situation **(C)**. Especially, the lose-lose situation **(C)** in the experiment is caused by the impulse between the green ball and the yellow ball.

In this paper, by clustering interaction forces (interaction states) using Self-organizing Maps, we developed a state transition model that considers basic scenarios of the system, i.e., the win-win situation, the kick-out effect, and the lose-lose competition (see Figure 2). In a four-ball system, a win-win situation indicates that all four balls stay in the vertical airflow; a kick-out effect means that one of the four balls falls out of the flow; a lose-lose competition refers to a situation where more than one ball falls out of the flow. Since the perturbation from the outside environment is negligible, all the collective behaviors result from the interactions among these balls through airflow or mechanical contact. We classified different collective behaviors observed in the experiment and simulator and figured out several probable causes of typical behaviors. Finally, the interaction state transfers during typical behaviors were investigated in the form of Markov chains, which revealed that certain time series of interaction states probably lead to typical collective behaviors.

## 2 Materials and methods

### 2.1 Collective Bernoulli-ball simulator

**FIGURE 3:**
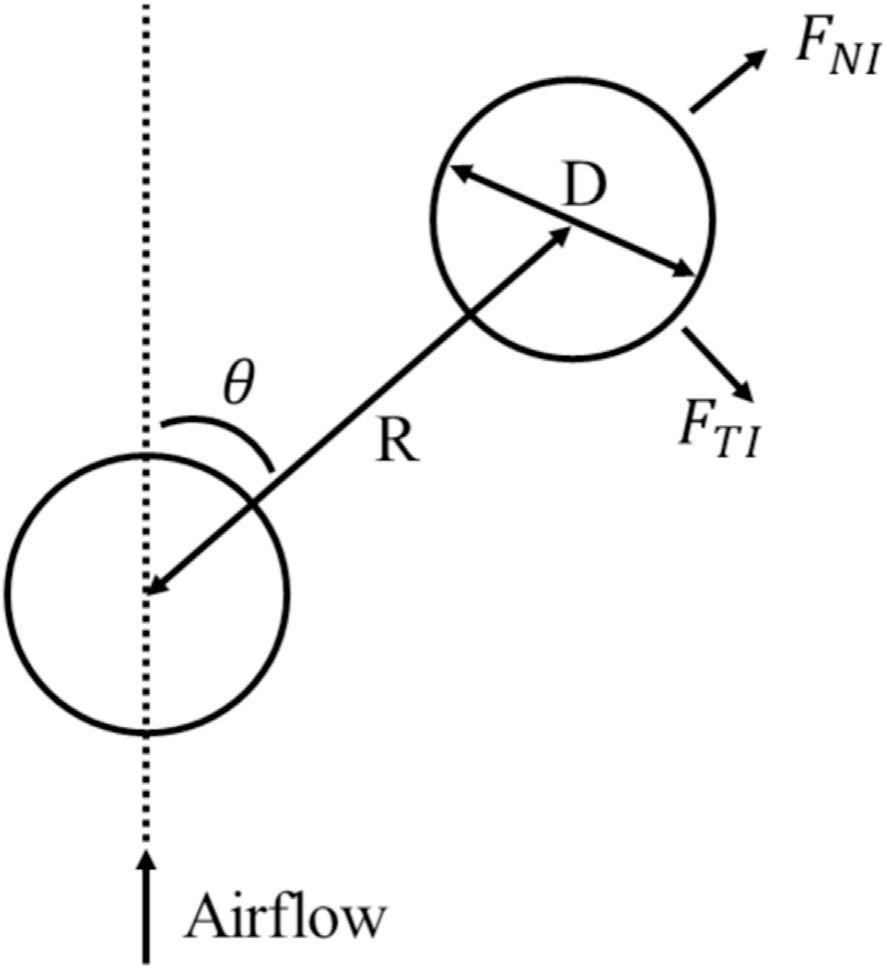
The interaction model of the collective Bernoulli-ball system in simulation. *F*_*NI*_ is the normal interaction force, and *F*_*TI*_ is the tangential interaction force between two balls. *θ* is the angle of attack of two balls, *D* is the diameter of the ball and *R* is the distance between two balls. The interaction forces depend on *θ* and *R*/*D*.

The collective Bernoulli-ball simulator consists of three parts. The first part focus on the forces that the flow field applies to the balls. The upward airflow provides a drag force that counters the weight of the ball. Horizontally the airflow is fast at the center and slows down with the distance from it. According to Bernoulli’s principle, a low-pressure area develops in the center and produces horizontal forces attracting the balls to the center. The second part takes the flow turbulence into account. The turbulence results in uncertain forces in the collective Bernoulli-ball system and was modeled as a Gaussian random process. The third part considers the interactions among the Bernoulli-balls, including the mechanical contact forces and the interaction forces through the airflow, shown in Figure 3. We investigate the collective behavior of four balls in the collective Bernoulli-ball system, and the error of the simulator could be minimized to 2.00% in position trajectory compared to the real-world experiment. After 10 simulations with different random seeds, 90,000 frames of ball trajectories and the dataset interaction forces were collected. The simulator is implemented in the MATLAB SIMULINK environment. Example code, data and experiment video are available at https://github.com/Kyushudy/collective_bernoulli_ball_behavior_analysis.

### 2.2 Force cluster

Since all four balls share the same characteristics like diameters and weights, it is important to distinguish these balls by a dynamic property except their colors. For example, the behavior of the red ball falling means the same as the green ball falling, the blue ball falling, etc. By introducing the interaction forces, we can tell the magnitude of pushing (positive normal interaction forces *F*_*NI*_) or pulling (negative normal interaction forces) forces between every two balls. If one ball pulls all the other balls, it tries to bond with others, so we manually define it as a strong ball. In contrast, if one ball is pushed by all other balls, it gets repulsed by others, so we define it as a weak ball. Therefore, the strength of a ball is defined as follows:

$$Kb_{i}=\overset{4}{\underset{j=1}{\sum }}Fb_{ij},\;\;\;\;j\neq i,\;i=1,2,3,4,$$ (1)

where *Kb*_*i*_ is the strength of ball *i*, variable *j* indicates all other balls except ball *i*, and *Fb*_*ij*_ is the normal interaction force between ball *i* and *j* projected onto the line of their center of mass. For example, for ball 2, *Kb*_2_ is the summation of *Fb*_21_, *Fb*_23_, and *Fb*_24_. A smaller *Kb*_*i*_ indicates that ball *i* is stronger. By ranking *Kb*_*i*_ at every frame, we can sort the balls from the strongest ball to the weakest ball, labeled as the first (the strongest), second, third, and fourth (the weakest) ball. The sort of balls represents the dynamic property of every ball at every frame and may change as interaction forces change in different frames. After sorting, the strength of the *i*th ball is rewritten as follows:

$$K_{i}=\overset{4}{\underset{j=1}{\sum }}F_{ij},\;\;\;\;j\neq i,\;i=1,2,3,4,$$ (2)

where *K*_*i*_ is the strength of the *i*th ball, variable *j* indicates all other balls except ball *i*, and *F*_*ij*_ is the normal interaction force between the *i*th and *j*th ball projected onto the line of their center of mass.

As we have the sorted interaction forces *F*_*ij*_ at every frame, a self-organizing map (SOM) method is used to distinguish different frames based on their different combination of sorted interaction forces. After 200 iterations, the SOM clustered 90,000 frames into six states, and every state represents a typical combination of interaction forces, labeled from *S*_1_ to *S*_6_.

### 2.3 Behavior modality

The collective behaviors of the collective Bernoulli-ball system are generally classified into the win-win situation, the kick-out effect, and the lose-lose competition through the observation of the experiment and simulation. Through simulation, we found that these behaviors mainly result from two kinds of forces: the interaction forces between every two balls through airflow, and the impulse between two balls through mechanical contact. In this case, seven typical behaviors are defined as follows:

1. The win-win situation: All the four balls float in the airflow in dynamic stability and no one falls, labeled as *B*_1_.
2. The kick-out effect: One ball falls out of the airflow and all the other three balls are inside the airflow.
  a. There is no obvious cause for this kick-out effect, labeled as *B*_2_.
  b. The kick-out effect is caused by interaction forces: from 0.5 s before this behavior to the end of this behavior, there is at least one frame that the falling ball is pushed by all the other three balls, labeled as *B*_3_.
  c. The kick-out effect is caused by impulses: from 0.5 s before this behavior to the end of this behavior, there is at least one frame that the falling ball has mechanical contact with any other balls, labeled as *B*_4_.
3. The lose-lose competition: More than one ball falls out of the airflow.
  a. There is no obvious cause for this lose-lose competition, labeled as *B*_5_.
  b. The lose-lose competition is caused by interaction forces: From 0.5 s before this behavior to the end of this behavior, there is at least one frame that at least one falling ball is pushed by all the other three balls, labeled as *B*_6_.
  c. The lose-lose competition is caused by impulses: From 0.5 s before this behavior to the end of this behavior, there is at least one frame that at least one falling ball has mechanical contact with any other balls, labeled as *B*_7_.

## 3 Results

### 3.1 States of interaction forces

**FIGURE 4:**
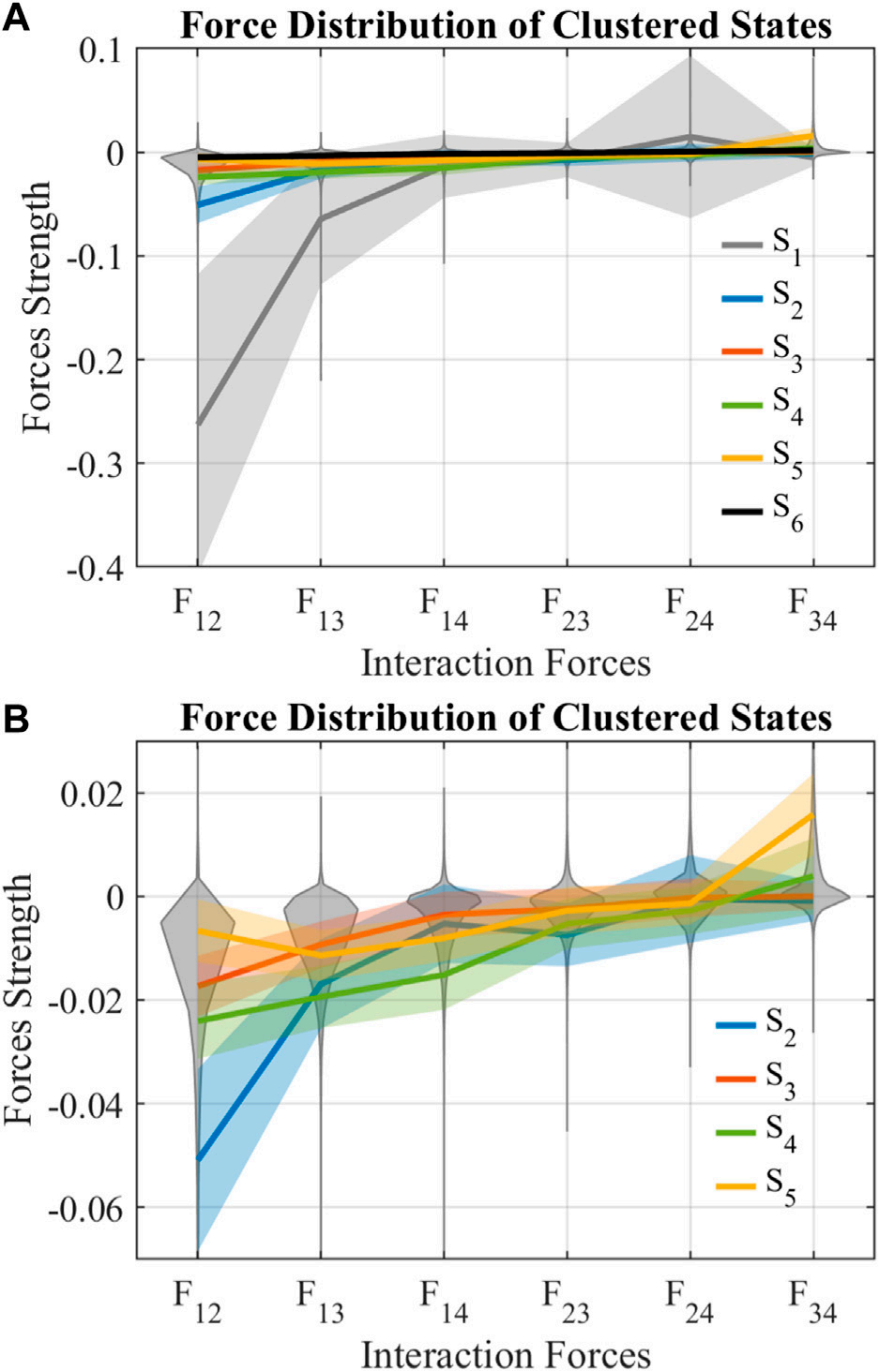
Force distribution of clustered states. The gray violin plot shows the distribution of interaction forces. The lines show the mean of interaction forces in respective states, and the shadings around the mean show its standard deviations.

The SOM clustered six states of combination of interaction forces *F*_12_, … , *F*_34_, labeled from *S*_1_ to *S*_6_. As shown in Figure 4A, *S*_1_ consists almost all the outliers of *F*_12_, *F*_13_, *F*_14_, and *F*_24_, indicating that *S*_1_ is the outlier of all the states. *S*_6_ consists of all the interaction forces near 0, indicating that there are few interactions between balls in *S*_6_. Figure 4B zooms in and shows details of *S*_2_ to *S*_5_. *S*_2_ consists of a low region of *F*_12_ while other forces are relatively near 0, indicating that the first ball and the second ball strongly bond together in *S*_2_. *S*_3_ consists of all interaction forces smaller than those of *S*_2_, indicating that *S*_3_ is a weaker version of *S*_2_, and the bond between balls in *S*_3_ is not strong. *S*_4_ consists of similarly low *F*_12_, *F*_13_, and *F*_14_, while other forces are near 0, which means that the first ball tries to catch all the other balls in *S*_4_. *S*_5_ consists of high *F*_34_, indicating that the third ball tries to move away from the fourth ball.

**FIGURE 5:**
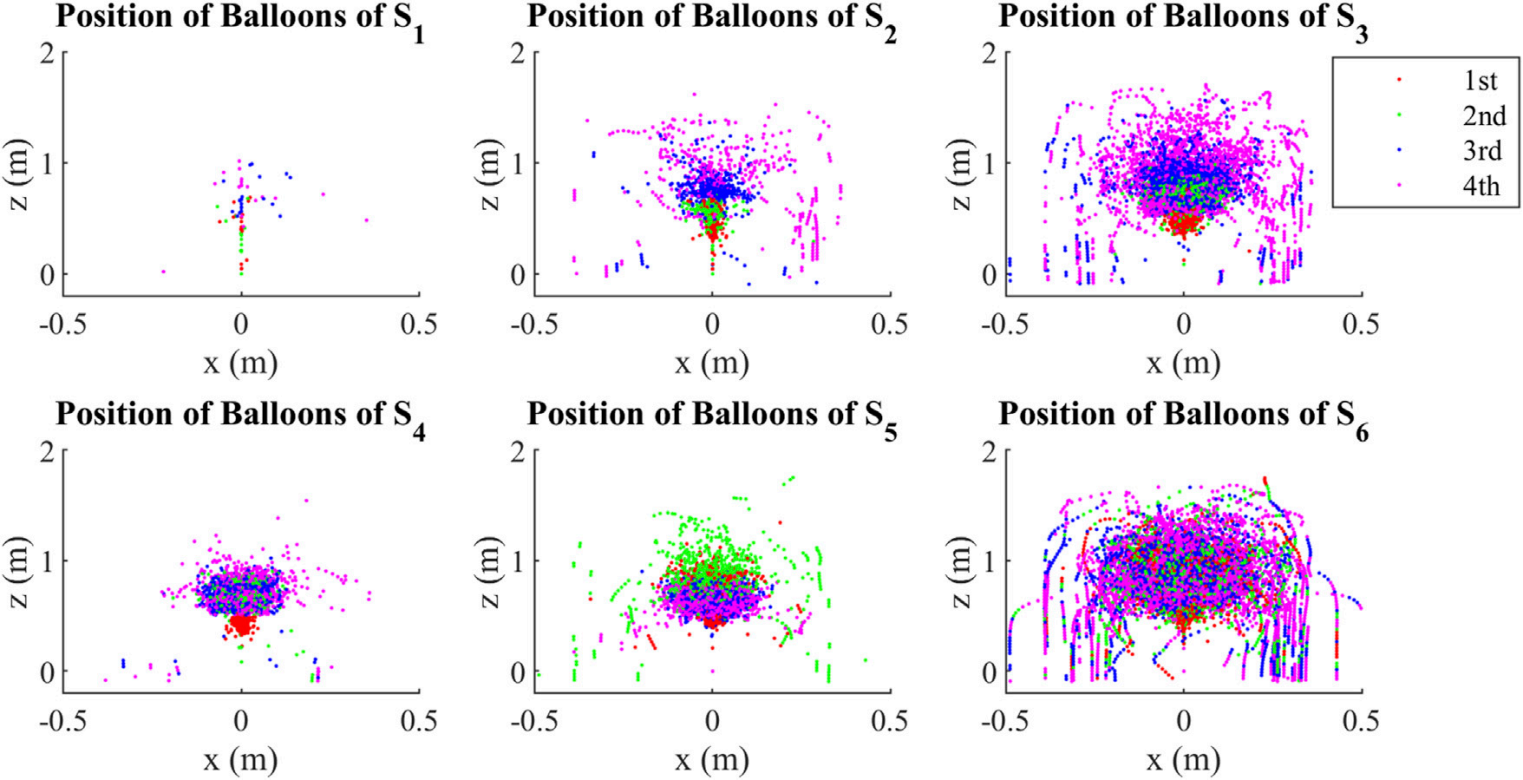
Position distribution of clustered states. Every dot shows the position of a ball at a frame. The density of dots shows the frequencies of clustered interaction states, and the distribution of dots in different colors shows the distribution of all the four balls from the strongest (first) to the weakest (fourth).

The characteristics of states can be shown more clearly in a position-based plot. Although states were clustered through interaction forces, they show typical ball position distribution in Figure 5. Since the density of dots represents the number of occurrences of a state, we found that *S*_1_ rarely happens in the collective Bernoulli-ball system, while *S*_6_ happens most frequently. *S*_2_ and *S*_4_ happen in medium frequencies while *S*_3_ and *S*_5_ happen in relatively higher frequencies. In *S*_1_, balls are distributed in a vertical line. In *S*_2_ and *S*_3_, there are clear layer distributions of balls: the first, second, third, and fourth ball are distributed in a vertical sequence from the bottom to the top, and the balls in *S*_3_ distribute wider than those in *S*_2_. In *S*_4_, the first ball is at the bottom, the second and third ball share the middle region, and the fourth ball is above them. In *S*_5_, the first ball, the third ball, and the fourth ball share the bottom, while the second ball is at the top. In *S*_6_, all balls distribute widely in similar distribution. The first ball is not likely to fall in any state. The second ball is most likely to fall in *S*_5_ and *S*_6_. The third ball and the fourth ball mostly fall in *S*_2_, *S*_3_, and *S*_6_.

### 3.2 Typical behaviors

Although we classified seven behaviors of the collective Bernoulli-ball system, some of them are stochastic and unexceptional. Therefore, we selected four typical behaviors to discuss their ball trajectories, interaction forces, and state transfer. These typical behaviors are the kick-out effect through interaction forces *B*_3_, the kick-out effect through impulses *B*_4_, the lose-lose competition through interaction forces *B*_6_, and the lose-lose competition through impulses *B*_7_. Notice that in this section we used unsorted forces *Fb*_*ij*_ and label the red, green, blue, and yellow ball as the ball 1, 2, 3, 4, respectively. This helps us to correlate the ball trajectories with the interaction forces based on consistent ball labels.

#### 3.2.1 The kick-out effect through interaction forces

**FIGURE 6:**
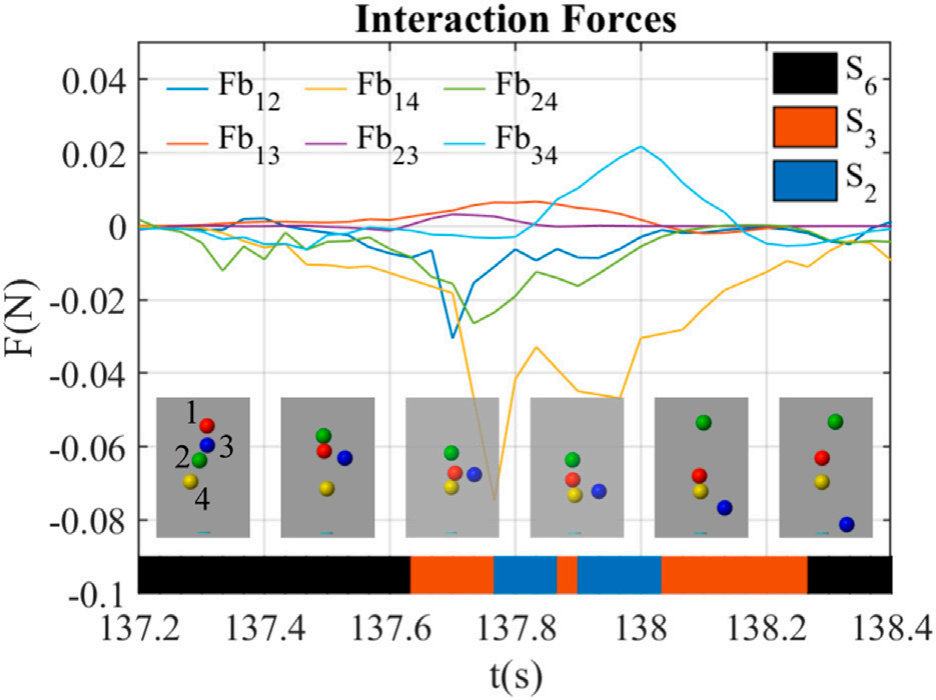
Interaction forces between balloons during kick-out behavior led by interaction forces (*B*_3_). The balls are labeled as red (1), green (2), blue (3), and yellow (4). The line chart shows the magnitude of interaction forces between balls. The frames show the exact positions of the balls at that time. The color bar shows the interaction states and their transfers in this period of time.

Figure 6 shows a kick-out effect happening at *t* = 137.2 ∼ 138.4 s. During this period, the blue ball (ball 3) was kicked out of the airflow, while other balls were staying inside. There were frames at around *t* = 137.83 s showing that the interaction forces of ball 3 (*Fb*_13_, *Fb*_23_, *Fb*_34_) were all positive, indicating that ball 3 was rejected by other three balls by the interaction forces through the airflow. At *t* = 137.3 s, all four balls were stable in the airflow. At *t* = 137.5 s, the blue ball was kicked out of the airflow due to positive *Fb*_13_ and *Fb*_23_. The blue ball tried to go back to the center because of the centering force based on Bernoulli’s principle, but *Fb*_13_, *Fb*_23_, *Fb*_34_ were increasingly positive that resisted the return of the blue ball at *t* = 137.5 ∼ 138.1 s. Afterward, the blue ball was completely outside the airflow and falled to the ground.

The interaction state shifted from *S*_6_ to *S*_3_ when entered this behavior in Figure 6, and from *S*_3_ to *S*_6_ when it exited. During *t* = 137.6 ∼ 138.2 s, the interaction state generally shifted from *S*_3_ to *S*_2_ and then back to *S*_3_.

#### 3.2.2 The kick-out effect through impulses

**FIGURE 7:**
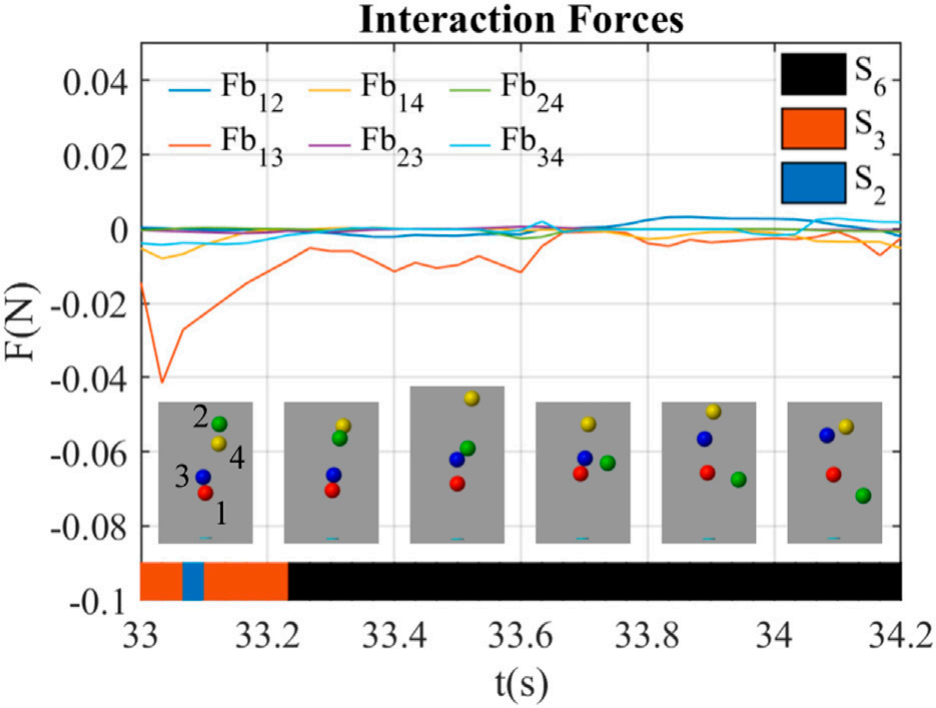
Interaction forces between balloons during kick-out behavior led by impulses (*B*_4_).

Figure 7 shows a kick-out effect happening at *t* = 33.0 ∼ 34.2 s. During this period, the green ball (ball 2) was kicked out of the airflow, while other balls were staying inside. At *t* = 33.5 s, the blue ball hit the green ball, which resulted in the fall of the green ball. The interaction forces were almost near 0 in this behavior except that *Fb*_13_ was negative during *t* = 33.0 ∼ 33.6 s, which indicated the red ball (ball 1) and the blue ball (ball 3) bond together in this period of time.

The interaction state in Figure 7 was always *S*_6_ before the impulse at *t* = 33.5 s till the end of the green ball’s fall at *t* = 34.2 s. This indicates that the impulse dominates this behavior while the interaction forces did not make remarkable changes to the system.

#### 3.2.3 The lose-lose competition through interaction forces

**FIGURE 8:**
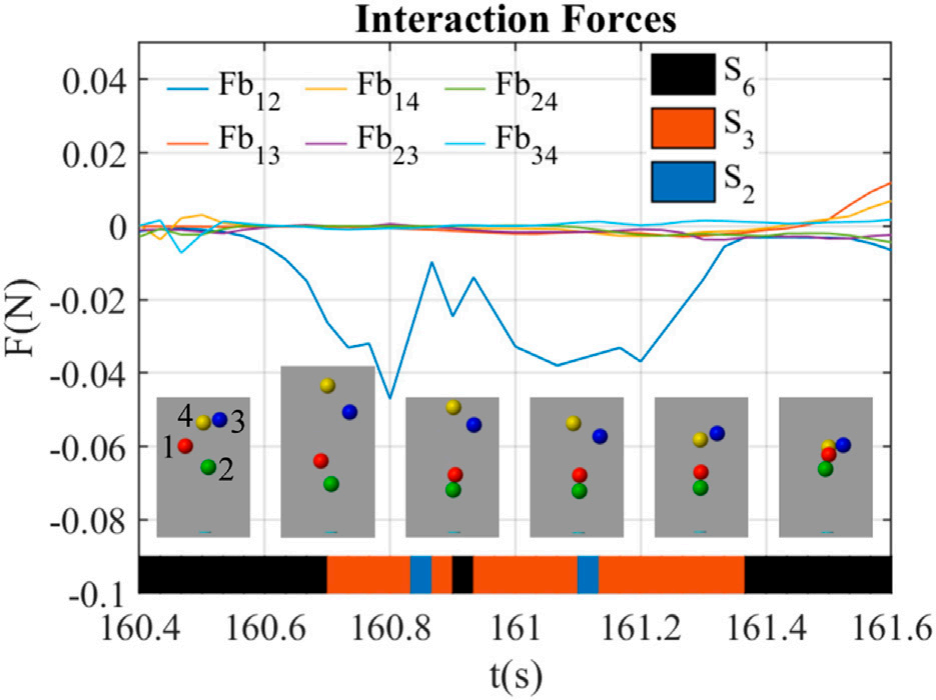
Interaction forces between balloons during lose-lose behavior led by interaction forces (*B*_6_).

Figure 8 shows a lose-lose competition happening at *t* = 160.4 ∼ 161.6 s. During this period, the blue ball (ball 3) and the yellow ball (ball 4) were out of the airflow, while other balls were staying inside. There were frames at around *t* = 160.6 s showing that the interaction forces of ball 4 (*Fb*_14_, *Fb*_24_, *Fb*_34_) were all positive, indicating that ball 4 was rejected by other three balls by the interaction forces through the airflow. At *t* = 160.5 s, the red ball, the blue ball, and the yellow ball were all out of the airflow, and the forces were all near 0. During *t* = 160.6 ∼ 161.3 s, *Fb*_12_ was negative and attracted the red ball (ball 1) back to the airflow, while all other interaction forces were slightly positive near 0, which kept the blue ball (ball 3) and the yellow ball (ball 4) outside of the airflow. At *t* = 161.3 s, *Fb*_34_ was clearly positive, which continuously kept the blue ball (ball 3) and the yellow ball (ball 4) outside. Afterward, the blue ball (ball 3) and the yellow ball (ball 4) went back to the airflow due to the centering forces.

The interaction state shifted from *S*_6_ to *S*_3_ when entered this behavior in Figure 8, and from *S*_3_ to *S*_6_ when it exited. During *t* = 160.7 ∼ 161.3 s, the interaction state was generally *S*_3_, but sometimes shifted to *S*_2_ and *S*_6_, and immediately back to *S*_3_.

#### 3.2.4 The lose-lose competition through impulses

**FIGURE 9:**
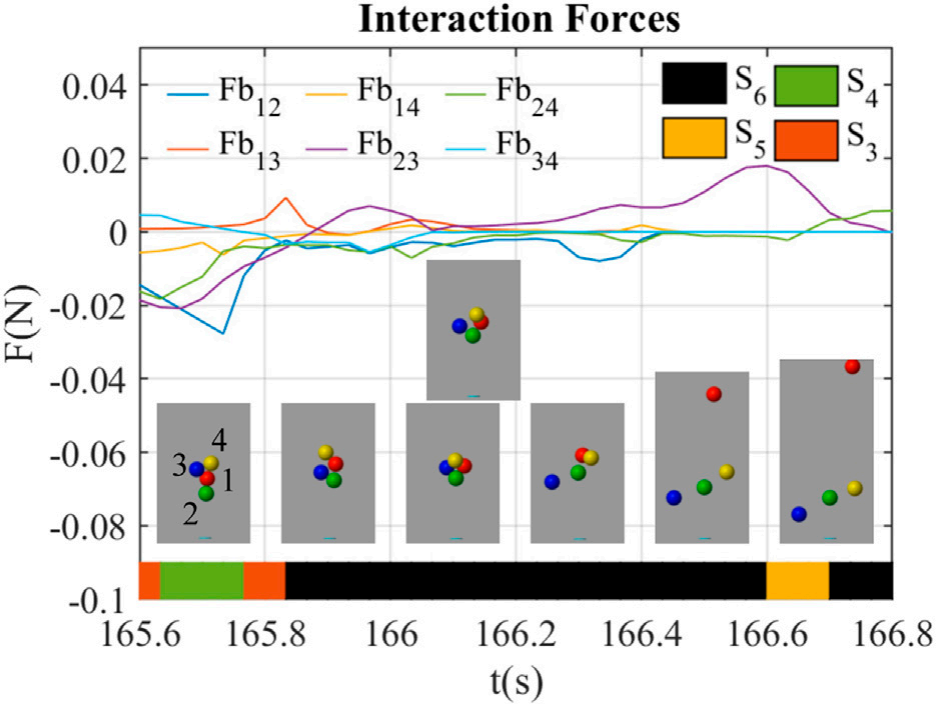
Interaction forces between balloons during lose-lose behavior led by impulses (*B*_7_).

Figure 9 shows a lose-lose competition happening at *t* = 165.6 ∼ 166.8 s. During this period, the red ball (ball 1), the blue ball (ball 3), and the yellow ball (ball 4) were out of the airflow, while the green ball (ball 2) was staying inside. At *t* = 166.1 s, the blue ball hit the yellow ball, which results in the fall of them both. At *t* = 166.2 s, the green ball hit the red ball, which kicked the red ball out of the airflow. The interaction forces were almost near 0 in this behavior except that *Fb*_23_ was positive during *t* = 165.9 ∼ 166.7 s, which indicated the green ball (ball 2) keeps the blue ball (ball 3) outside.

The interaction state in Figure 9 was generally *S*_6_ before the first impulse at *t* = 166.1 s till the end of the balls’ fall at *t* = 166.8 s. This indicates that the impulses dominate this behavior while the interaction forces did not make remarkable changes to the system.

### 3.3 State transfer in behaviors

**FIGURE 10:**
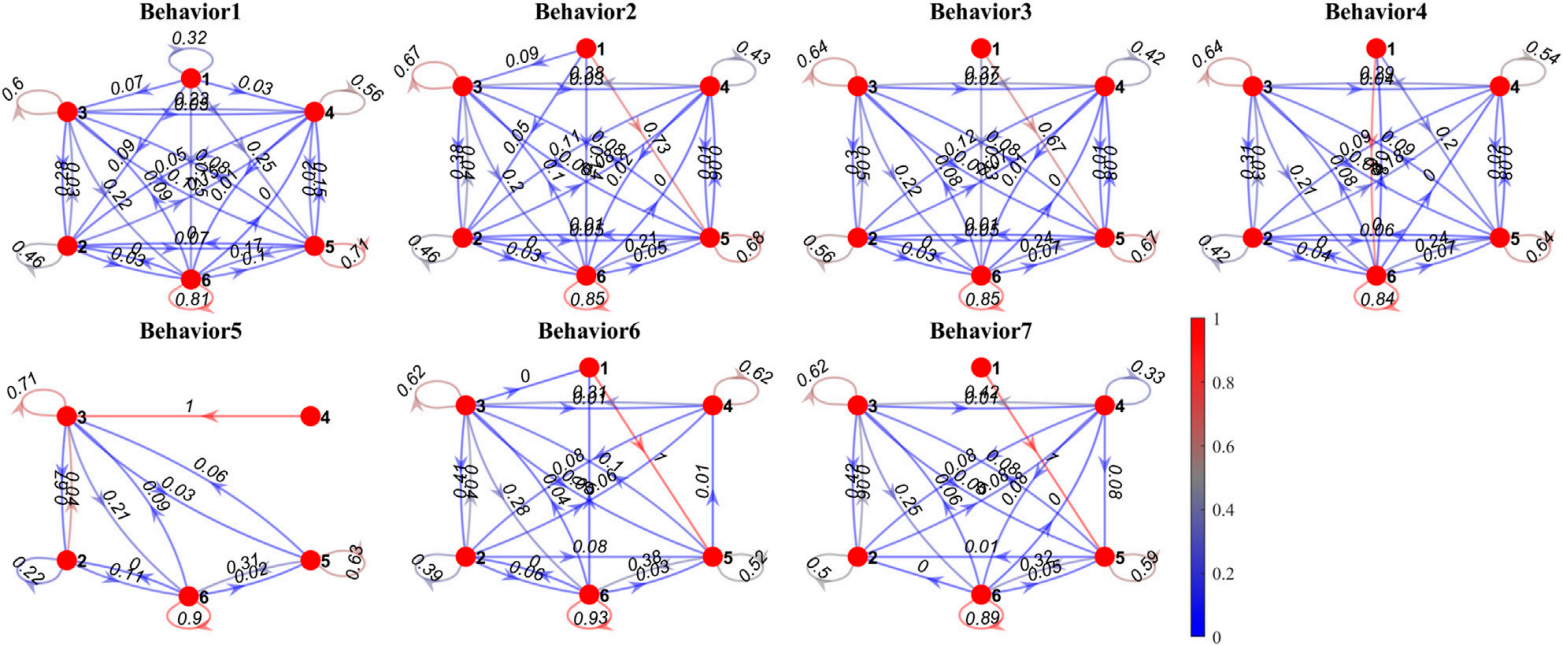
Markov chains of interaction state transfer in typical behaviors. The red dots indicate interaction states from *S*_1_ to *S*_6_. The color of the arrows indicates the probabilities of state transfer.

Figure 10 shows the state transfer model (Markov chains) of interaction forces leading to certain behavior (from *B*_1_ to *B*_7_). Some shared patterns between behaviors are discovered: state 1 mostly shifts to other states while other states rarely shift to state 1. The probability of staying in state 6 is always higher than 0.8, the probabilities of staying in state 3,4,5 are around 0.6, and the probability of staying in state 2 is about 0.4. The transfer rates from state 2 or state 4 to state 3 are around 0.3. The transfer rates from state 3 or state 5 to state 6 are around 0.2, while the transfer rates from state 6 to state 3 or state 5 are around 0.1. The transfer rates between state 2 and state 4 are about 0.1, and similarly, the transfer rates between state 3 and state 5 are about 0.1. Other transfer rates are mostly below 0.1.

In particular, the probability of staying in state 6 increases from *B*_1_ (0.81) to *B*_7_ (0.89), indicating that the more balls fall, the more probably the system stay in state 6. Similarly, the transfer rate from state 5 to state 6 increases from *B*_1_ (0.17) to *B*_7_ (0.32), the transfer rate from state 2 to state 3 increases from *B*_1_ (0.28) to *B*_7_ (0.42), the transfer rate from state 4 to state 3 in *B*_2_ (0.38) to *B*_7_ (0.42) is higher than that in *B*_1_ (0.23), and the transfer rates from state 3 or state 5 to state 6 increase from *B*_1_ to *B*_7_, while the transfer rates from state 6 to state 3 or state 5 decrease from *B*_1_ to *B*_7_.

**FIGURE 11:**
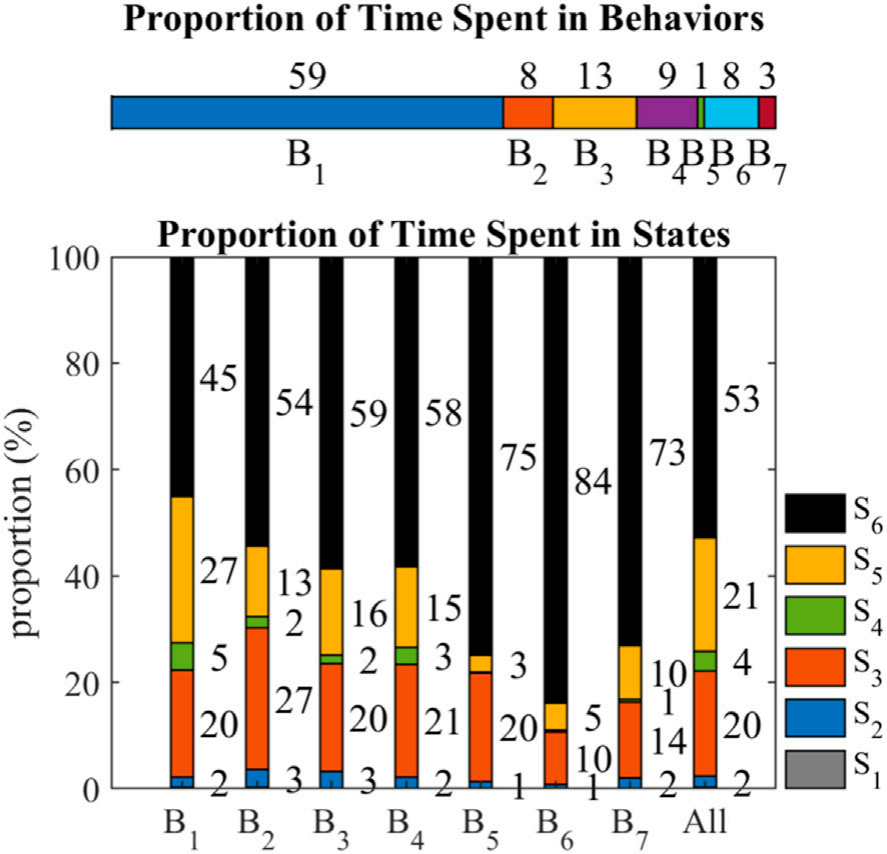
The proportion of time spent in behaviors and states. The values near the stacked bars indicate the proportion of time in percentage, and values less than 1% are hidden in this graph.

Figure 11 shows the proportions of time spent in behaviors and states. *B*_1_ (59%) takes up half of the time. *B*_2_ (8%), *B*_3_ (13%), *B*_4_ (9%), and *B*_6_ (8%) take about 10% of the time, while *B*_5_ (1%) and *B*_7_ (3%) rarely happen in the collective Bernoulli-ball system. Among all the frames, *S*_6_ (53%) takes up half of the time. *S*_5_ (21%) and *S*_3_ (20%) take about 20% of the time, while *S*_4_ (4%), *S*_2_ (2%) and $S_{1}\;\left(<1\%\right)$ rarely happen in the collective Bernoulli-ball system.

Compared with the overall proportion, *B*_1_ has less proportion of *S*_6_ (45%) and more proportion of *S*_5_ (27%). The proportion of *S*_6_ increases from *B*_1_ (45%) to *B*_7_ (73%), and the proportion of *S*_5_ decreases from *B*_1_ (27%) to *B*_7_ (10%). The proportion of *S*_4_ is 5% in *B*_1_, around 2% in *B*_2_, *B*_3_, and *B*_4_, and less than 1% in *B*_5_, *B*_6_, and *B*_7_. The proportion of *S*_3_ is always around 20% among behaviors except *B*_2_ (27%), *B*_6_ (10%), and *B*_7_ (14%). The proportions of *S*_2_ (2%) and $S_{1}\;\left(<1\%\right)$ are similar among all the behaviors.

## 4 Discussion

The results show that the interaction of balls depends on their relative positions, and the sequence of interaction states reflects collective behaviors. Although we clustered the interaction states through the properties of interaction forces, the position distribution from the strongest ball to the weakest ball in the interaction states seems to have a clear layer as shown in Figure 5. This suggests that although the balls with different colors are equal, their strength, defined by the interaction forces that they provide to other balls, distinguishes them. Thus the interaction state in the case with various strength balls can be represented by their positions. In addition, Figure 5 shows that balls are symmetrically distributed around the vertical axis. Therefore, the position of the ball can be represented by a reduced vector of the ball’s altitude and horizontal distance from the center of the flow. Consequently, collective behavior can be determined by relating interaction forces and relative strengths among balls to the reduced vector of position. For example, Figure 6 shows a kick-out behavior where the red, green, and yellow balls are pushing the blue ball out of the air stream. During this typical behavior from *t* = 137.5 s to *t* = 138.1 s, the position of the green, red, and yellow balls were distributed horizontally in the canter of the flow for various heights, while the position of the blue ball was far from the flow center. The force trajectories show that when the blue ball was vertically close to the green and red balls from *t* = 137.5 s to *t* = 137.8 s, the interaction forces (*Fb*_13_, *Fb*_23_) between them became positive and increased to repulse the blue ball. When the blue ball was vertically far from the green and red balls but close to the yellow ball from *t* = 137.8 s to *t* = 137.9 s, *Fb*_13_, *Fb*_23_ decreased to around 0, while *Fb*_34_ became positive and increased to repulse the blue ball. This effect made the green ball the weakest ball since the interaction forces (*Fb*_13_, *Fb*_23_, *Fb*_34_) related to it were all higher than other forces. Therefore, as the weakest blue ball fell, the interaction state switched in a chain (*S*_6_ − *S*_3_ − *S*_2_ − *S*_3_ − *S*_6_) and consequently led to a kick-out behavior.

Behaviors spent minor time on *S*_1_ and it has high possibilities to switch to other states, indicating that *S*_1_ is an outlier of the states, and it always immediately switches to other states. Mostly, behaviors spent the time on *S*_6_, and the probability of staying at *S*_6_ is also pretty high, indicating *S*_6_ is a base state of all behaviors, and the state transfer from *S*_6_ to other states determines its behavior. As shown in Figures 10, 11, if *S*_6_ is more likely to shift to *S*_5_ or *S*_3_, the behavior will more likely be *B*_1_, a win-win situation. If *S*_6_ is slightly less likely to shift to *S*_5_, and the proportion of *S*_4_ decreases, the behavior will more likely be *B*_2_, *B*_3_, and *B*_4_, a kick-out effect. If *S*_6_ is more likely to keep unchanged, and the proportion of all other states decreases, the behavior will more likely be *B*_4_, *B*_5_, and *B*_6_, a lose-lose competition.

If we simplify the interaction forces to pushing (positive forces) and pulling (negative forces), and regard these pushing and pulling forces as kinds of competition and collaboration among these balls, a more interesting discovery is: a certain time sequence of competition and collaboration patterns leads to certain behaviors of the collective Bernoulli-ball system. Additionally, the Bernoulli-ball system can be considered to be autonomous, as the collective behavior is determined by the set of local rules (physics principles) related to a flow field with no leader or external controller. Since the interaction happens through the medium of the airflow, the noise from the environment (e.g., turbulence) introduces the stochastic effect to the system. Therefore, the environment of our system in a way affects the behavior of the balls and can be potentially used for control, which makes the Bernoulli-ball system applicable to swarm robotics.

The collective Bernoulli-ball system can be also used to analyze other multi-agent systems like the Brownian motion (Kramers, 1940) or complex systems like the falling paper (Howison et al., 2020b). These systems exhibit similar behaviors to the Bernoulli-ball system since they are autonomous, rely on the body-environment interaction (the fluid in the Brownian motion and the airflow in the falling paper experiment), depend on local interaction rules, and have a certain level of randomness. Furthermore, the control of airflow in the collective Bernoulli system makes it more appealing for practical use in swarm robotics.

## 5 Conclusion

In this paper, we developed a state-transfer model that describes the collective behavior of the multi-ball Bernoulli system. To convert interaction forces into behavior states, we employed self-organizing maps to find distinct clusters of interaction forces. We defined seven various behaviors that consider both cooperation and competition and applied the Markov chain model, with six interaction force states, to find the correlation between the interaction states and collective behaviors in this system. In general, the more time this system spends on organized interaction states (*S*_6_, *S*_5_, and *S*_4_), the more probable this system will be performing a self-organized behavior (*B*_1_).

In the future, we will focus on the development of control methods to keep this collective Bernoulli-ball system in defined interaction states. Additionally, it is interesting to see how this system reacts to external cues, e.g., the fluid speed, the perturbation of the surrounding airflow, and the external object that obstruct the fluid. By finding the efficient input method for control of the Bernoulli-ball system, we can try to apply it for physical computing (Fresco, 2014; Piccinini, 2015) and as a reservoir computer like the liquid state machine (Maass et al., 2002).

## Data Availability

The datasets presented in this study can be found in online repositories. The names of the repository/repositories and accession number(s) can be found below: https://github.com/Kyushudy/collective_bernoulli_ball_behavior_analysis.
